# Pilot Study of Immunoblots with Recombinant *Borrelia burgdorferi* Antigens for Laboratory Diagnosis of Lyme Disease

**DOI:** 10.3390/healthcare6030099

**Published:** 2018-08-14

**Authors:** Song Liu, Iris Du Cruz, Catherine Calalo Ramos, Paula Taleon, Ranjan Ramasamy, Jyotsna Shah

**Affiliations:** 1 ID-FISH Technology Inc., 797 San Antonio Road, Palo Alto, CA 94303, USA; steells9508@gmail.com; 2 IGeneX Inc., 795 San Antonio Road, Palo Alto, CA 94303, USA; icruz@igenex.com (I.D.C.); cramos@igenex.com (C.C.R.); paulataleon@igenex.com (P.T.); rjr200911@yahoo.com (R.R.)

**Keywords:** *Borrelia burgdorferi*, immunoblot, laboratory diagnosis, Lyme disease, tick-borne diseases, western blot

## Abstract

Accurate laboratory diagnosis of Lyme disease (Lyme borreliosis), caused by the spirochete *Borrelia burgdorferi* (BB), is difficult and yet important to prevent serious disease. The US Centers for Disease Control and Prevention (CDC) presently recommends a screening test for serum antibodies followed by confirmation with a more specific Western blot (WB) test to detect IgG and IgM antibodies against antigens in whole cell lysates of BB. *Borrelia* species related to BB cause tick-borne relapsing fever (TBRF). TBRF is increasingly recognized as a health problem in the US and occurs in areas where Lyme disease is prevalent. The two groups of *Borrelia* share related antigens. We have developed a modified WB procedure termed the Lyme immunoblots (IBs) using recombinant antigens from common strains and species of the BB sensu lato complex for serological diagnosis of Lyme disease. A reference collection of 178 sera from 26 patients with and 152 patients without Lyme disease were assessed by WB and IB in a blinded manner using either criteria for positive antibody reactions recommended by the CDC or criteria developed in-house. The sensitivity, specificity, positive and negative predictive values obtained with the reference sera suggest that the Lyme IB is superior to the Lyme WB for detection of specific antibodies in Lyme disease. The Lyme IB showed no significant reaction with rabbit antisera produced against two *Borrelia* species causing TBRF in the US, suggesting that the Lyme IB may be also useful for excluding TBRF.

## 1. Introduction

Lyme disease (Lyme borreliosis) is the most common tick-borne disease in North America and Europe and one where accurate diagnosis is difficult and yet important for initiating treatment to prevent progression to more severe disease [1,2,3]. Lyme disease is caused by spirochete bacteria of the *Borrelia burgdorferi* sensu lato (BBsl) complex. Globally, an increasing number of species are being characterized within the BBsl complex. *Borrelia burgdorferi* sensu stricto (BBss) is principally responsible for human infections in the US [3,4], with known contributions from *B. bissettii* [5] and *B. mayonii* [6]. *Borrelia afzelii*, *B. garinii*, and BBss are important causes of human infections in Europe and Asia [7]. Although 26,203 confirmed and 10,226 probable cases of Lyme disease were reported in the US in 2016, it is estimated that the actual number of people diagnosed with Lyme disease is more likely over 300,000, making it the most common vector-borne disease in the country according to the Centers for Disease Control and Prevention (CDC) [8].

The main vectors that transmit BBsl in the US are the blacklegged or deer tick, *Ixodes scapularis*, in northeastern, mid-Atlantic, and northcentral US, and *Ixodes pacificus* on the West coast. A characteristic ‘bulls-eye’ Erythema Migrans (EM) rash that results from an infected tick bite feed is generally considered to be the earliest and best indicator of acute BBsl infection, but EM may be absent or go unrecognized in 20–40% of patients [9,10]. If the initial infection is not treated, patients can progress to disseminated Lyme disease that may be characterized by cardiac, musculoskeletal, and neurological manifestations [10]. Symptomatic clinical diagnosis in late stages of the disease can be difficult when a history of EM rash and tick bite may be lacking, as symptoms are shared with several other diseases [10,11,12,13,14,15,16]. A separate group of *Borrelia* species, the relapsing fever group (e.g., *Borrelia hermsii*, *B. coriaceae* and *B. miyamotoi*), cause tick-borne relapsing fever (TBRF) and some are transmitted by the same *Ixodes* species that transmit Lyme borreliosis [15,16]. TBRF is becoming increasingly recognized as a health problem in the US and shares some clinical symptoms with Lyme disease [15,16]. There are many cross-reacting as well as group-specific antigens in the two groups of *Borrelia* [15,16], making the serological differentiation of Lyme disease and TBRF an important need. 

Detection of BBsl using microscopy, culture, nucleic acid amplification and antigen detection have limited sensitivity and specificity, except in early infections with an EM rash [10,17,18,19], where PCR assay on skin biopsy is a sensitive diagnostic method [19]. PCR-based detection in blood is relatively insensitive for detecting late stage-Lyme disease because BBsl tends to leave the bloodstream and sequester itself in tissues. However PCR assays on synovial fluid for Lyme arthritis and cerebrospinal fluid for neuroborreliosis are reportedly useful diagnostic procedures [20,21,22]. Urine is PCR tested in Lyme disease less frequently because of poor reproducibility and the presence of PCR inhibitors—a problem shared with blood samples [23,24], but a recently described Lyme Multiplex PCR-dot blot assay overcomes this shortcoming [25]. 

The laboratory diagnosis of Lyme disease therefore mainly depends presently on a CDC recommended two-tiered serological testing system to detect specific antibodies in patient sera. In this system, a sensitive Enzyme-linked Immunosorbent Assay (EIA) or Immunofluorescent Antibody (IFA) test is performed as a screen, followed by a more specific Western Blot (WB) on whole cell lysate of BBsl for confirmation if the result obtained by EIA or IFA is indeterminate or positive [10,17,18]. The CDC guidelines for positivity in IgG and IgM WBs for evidence of antibodies against the Lyme disease bacteria [10,17] are listed in Section 2.6 below. They have been the standard for WB interpretation since the Dearborn conference of 1995. Two-tiered serological testing has a reported sensitivity of 30–40% during the first week after developing an EM rash and 29–78% in convalescent stages after treatment [11,26]. BBsl have evolved mechanisms to subvert host immunity [25,27] and seronegativity has been observed in late Lyme disease [25,28]. Pathogens that cause diseases such as Anaplasmosis, Babesiosis, Ehrlichiosis and TBRF borreliosis are also transmitted by the same ticks that transmits BBsl. Patients with Lyme disease can therefore harbor other tick-borne pathogens and hence it is important to detect and differentiate BBsl-specific antibodies in such possible cases of multiple infections [14,15,29,30,31,32]. False positive results in detecting IgM and IgG antibodies against BBss have also been reported in patients with rheumatoid arthritis, infectious mononucleosis, autoimmune diseases, bacterial endocarditis, syphilis, other spirochetal infections and *Helicobacter pylori* infections [33]. Different criteria have been developed in-house for positive anti-BB antibody reactions in IgG and IgM WB [34] and these are described in Section 2.6 below. 

The expression of specific BBss protein antigens is dependent on culture conditions, growth phase and genetic variation between strains of BBss [12]. WB assay sensitivity in Lyme disease improves when antigens from more than one strain are used [34,35,36]. Tick-borne BB infection can take place anywhere in the world due to international travel. In order to detect Lyme disease acquired abroad, a WB antigen panel from different BBsl species is expected to be more useful. However a WB panel employing cell lysates that include all common BBsl species would be expensive and impractical for clinical tests. Furthermore, sensitivity can be affected by differential expression of target antigens. Additionally, when whole cell lysate proteins are separated only by size during SDS gel electrophoresis, multiple proteins of similar size may appear to migrate together causing reduced specificity [34]. 

Purified recombinant antigens are increasingly being used in many laboratories in a variety of test formats, including EIA and WB, to detect IgG and IgM antibodies to BBsl and BBss for diagnosis [37,38]. To overcome some of the drawbacks associated with WB-based diagnosis, we recently developed IgM and IgG WBs that use a panel of purified recombinant protein antigens from several species of the BBsl complex for identifying the presence of antibodies to the different antigens. These WBs that use purified recombinant antigens as targets are termed Lyme Immunoblots (IBs) here to differentiate them from Lyme WBs that use whole cell lysates as target antigens. The recombinant antigens we used in the Lyme IBs represent all the antigens used for characterizing positive WB reactions in the current in-house [34] and CDC guidelines [10,17]. We investigated the comparative diagnostic performance of Lyme IBs and WBs with a set of well-characterized reference patient sera and also the reaction of antisera produced against two common TBRF *Borrelia* species in Lyme IBs.

## 2. Materials and Methods

### 2.1. Reference Sera

A total of 178 reference serum samples, including 26 sera from patients with confirmed Lyme disease, were tested. Details of the reference serum samples and test results provided by the suppliers are shown in Table 1. Out of 46 samples with antibodies to viruses provided by New York Biologics, 11 had antibodies to cytomegalovirus; 24 to Epstein-Barr virus; seven to herpes simplex virus; and four to hepatitis C virus. Once received, the sera were stored at 4 °C up to one week and at −20 °C for longer storage. Testing of reference sera was performed by laboratory personnel without prior knowledge of the expected results in the same manner as clinical samples from Lyme disease patients. The CDC provided EIA results, details of the bands that reacted in WBs for CDC Set 1 and 2 reference samples, WB interpretation by CDC criteria and two-tiered testing results. These samples were interpreted by in-house criteria too, for positivity as described in Section 2.6. EIAs and WBs were therefore not performed in-house on these 42 CDC samples, and only IBs were performed. On the remaining 136 samples, EIAs, WBs and IBs were performed. 

### 2.2. Detection of IgG and IgM Serum Antibodies to B. burgdorferi by *EIA*

Serum samples were tested by MarDX *B. burgdorferi* EIA (IgG, IgM) Test System as per the manufacturer’s product insert (MarDx Diagnostic Company, Trinity Biotec, Carlsbad, CA, USA). The MarDx test is an indirect EIA technique utilizing sonicated whole cell lysate antigens of *B. burgdorferi* (Strain B31) bound to polystyrene microwells, for detection of IgG and IgM antibodies to *B. burgdorferi*. 

### 2.3. Preparation of Antigen Strips for Lyme Western Blots

Nitrocellulose strips for WB were prepared from whole cell lysates of a mixture of the two BBss strains, B31 and 297, and used for WBs as previously described [34]. Briefly, proteins present in sonicated whole cell lysate were separated by acrylamide gel electrophoresis. The separated proteins were transferred onto nitrocellulose membrane (Amersham Protran, GE Healthcare Life Science). The membranes with bound proteins were washed in deionized water, blocked in 5% non-fat dry milk for one hour, dried and sliced into 3-mm strips.

### 2.4. Preparation of Antigen Strips for Lyme Immunoblots

Recombinant proteins derived from several US and European species of BBsl were used to prepare antigen strips for Lyme IBs. The recombinant proteins selected included all the proteins used in scoring WBs by the CDC and in-house criteria described in Section 2.6 below. P23 (OspC) and P31 (OspA) proteins from several different BBsl species were used as target antigens in the Lyme IB. Separate P39 (BmpA) antigens derived from European and US BBsl species were included in the panel of test antigens. Additionally, the variable surface antigen of BBss (VslE), and a hybrid protein containing the immunodominant region of VslE from different BBsl species termed C6 [39] were also used in the Lyme IBs as target antigens. Recombinant antigens were prepared by cloning the hybrid gene constructs or portions of the selected genes into pET vectors, and then expressing the proteins in *Escherichia coli* (GenScript, Piscataway, NJ, USA). The *E. coli*-produced recombinant BBsl proteins were then purified using metal affinity chromatography followed by gel filtration. All the recombinant proteins were >90% pure by Coomassie blue staining after SDS PAGE. 

Purified proteins and two control proteins, diluted to yield 7–19 ng of protein as a line in each 3 mm strip of membrane were sprayed in straight lines onto nitrocellulose membrane (Amersham Protran, GE Healthcare Life Science) using a BioDot liquid dispenser (BioDot, Irvine, CA, USA). The two control proteins were Protein L (Sigma, St. Louis, MO, USA) for detecting the addition of human serum (termed serum control), and a mixture of human IgM and IgG (Sigma, St. Louis, MO, USA) for detecting the addition of alkaline phosphatase conjugated anti-human antibodies (termed conjugate control). The membranes were then blocked with 5% non-fat dry milk and sliced into 3 mm wide strips.

### 2.5. Procedure for Detection of Borrelia Specific Antibodies on Lyme Immunoblots and Western Blots with Test Sera

Prior to use, each strip was labeled and then soaked in 1 mL of diluent (100 mM Tris, 0.9% NaCl, 0.1% Tween-20 and 1% non-fat dry milk) for 5 min in a trough. A 10 µL aliquot of the test or control serum was added to a corresponding IB or WB strip in the trough. The strips were then incubated at room temperature for one hour with serum, followed by three washes with wash buffer (KPL, Gaithersburg, MD, USA) at room temperature. After aspirating the final wash solution, strips for detecting IgG and IgM were incubated with alkaline phosphatase-conjugated goat anti-human IgG at 1:10,000 dilution and IgM at 1:6000 dilution respectively (KPL, Gaithersburg, MD, USA) for one hour. After three washes, bands were visualized by reaction with 5-bromo-4-chloro-3-indolylphosphatenitro-blue tetrazolium (BCIP/NBT, KPL, Gaithersburg, MD, USA). The reactions were terminated by washing with distilled water when a calibration control produced a visible band at 39 kDa. Alkaline phosphatase-conjugated rabbit antibody to the 39/93 kDa BBsl antigens (Strategic Biosciences, Stow, MA, USA) diluted in human serum was used as the calibration control as previously described [34]. Bands with lower intensity than the calibration control were reported as negative. The Lyme WB and IB strips were also reacted with a mixture of human sera from patients with confirmed Lyme disease as a positive control and sera from uninfected persons as a negative control. 

### 2.6. Scoring of Positive Serological Reactions 

The following antigen bands in kDa were scored on the Lyme IB and WB strips: for IgG, 18, 23 (OspC), 28, 30, 31 (OspA), 34 (OspB), 39 (BmpA), 41 (FlaB), 45, 58, 66 and 93; for IgM, 23 (OspC), 31 (Osp A), 39 (BmpA), 41 (FlaB), 93 and additionally band 34 (Osp B) for WBs. By the CDC criteria, IgM WB reactivity with two of the three antigen bands 23, 39 and 41 kDa or IgG WB reactivity with five of the ten antigen bands 18, 23, 28, 30, 39, 41, 45, 58, 66 and 93 kDa was considered a positive result for Lyme disease [10,17]. However, the CDC guidelines recommend that a positive IgM test is valid only for the first four weeks after infection, i.e., early Lyme disease, because false positive IgM reactions may develop in advanced Lyme disease [10,17]. By the in-house WB criteria, IgG and IgM WBs were considered positive if two from the following six antigens bands were present: 23, 31, 34, 39, 41 and 93 kDa, with the following exceptions: indeterminate if only bands of 31 and 41 kDa were present on IgG and/or IgM or 31 and 93 kDa on IgM; and an IgM WB was considered negative if only bands of 41 and 93 kDa were present. By the in-house criteria, an IgM IB was considered positive if two out of the four bands of 23, 31, 39 and 41 kDa were present, and an IgG IB was considered positive if two out of the six bands of 23, 31, 34, 39, 41 and 93 kDa were present. 

### 2.7. Lyme Immunoblots with Rabbit Antisera against Lyme and TBRF Borrelia Species 

Rabbit antisera were raised against whole cell lysates of the following BBsl species grown in culture: *B. burgdorferi* B31, *B. burgdorferi* 297, *B. afzelii*, *B. garinii*, *B. californiensis*, *B. spielmanii* and *B. valaisiana*. Rabbit antisera were also produced against whole cell lysates of the TBRF *Borrelia* species *B. hermsii* and *B. coriaceae*. These different immune rabbit antisera were used in Lyme IBs to investigate the cross-reactions with BBsl antigens. Antigen strips for Lyme IBs were prepared as described in Section 2.4 and reacted with different rabbit antisera essentially as described in Section 2.5 except for the use of individual *Borrelia* species- or strain-specific rabbit antisera in place of human sera as the primary antibody, followed by alkaline phosphatase-labelled goat anti-rabbit IgG (KPL, Gaithersburg, MD, USA) as the secondary antibody. Two additional strips were reacted with positive and negative control human sera as described in Section 2.5. 

### 2.8. Statistical Analysis 

The diagnostic sensitivity, specificity, positive predictive value (PPV), negative predictive value (NPV), their 95% confidence intervals and Fisher Exact Probability 2-tailed test (*p* values) were determined online [40]. 

## 3. Results

### 3.1. Lyme IgM and IgG Immunoblots

**Figure 1:**
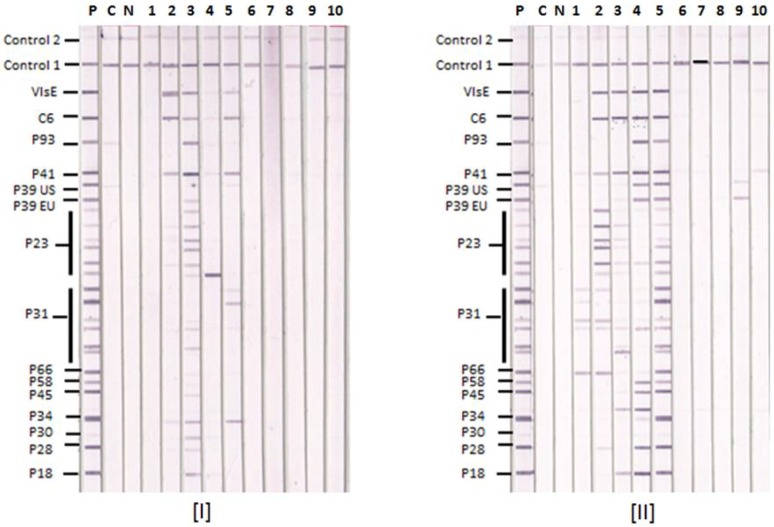
Lyme IgM and IgG Immunoblots with Serum Samples. Ten representative serum samples 1–10 were tested by [I] Lyme IgM and [II] Lyme IgG IBs. P—positive control, C—calibrator and N—negative control. Control 1—conjugate control, and Control 2—serum control. The positions of target antigens used in the IB strips are shown. P39 EU—P39 from European BBsl species, P39 US—P39 from US BBsl species.

Typical Lyme IgM and IgG IBs are illustrated with results from 10 sera in Figure 1. Sample 1 is positive only by the in-house criteria, samples 2–5 are positive by both CDC and in-house criteria and samples 6–10 are negative by both sets of criteria. The positive sera recognize P23 and/or P31 from different BBsl species in the IgG IBs. Three of the five positive sera recognize P39 from both US and European BBsl species in the IgG IBs. Four of the five positive sera also recognized VlsE and C6. 

### 3.2. Comparative Reactions of Four Sera in Lyme IgM Western Blots and Lyme IgM Immunoblots

**Figure 2:**
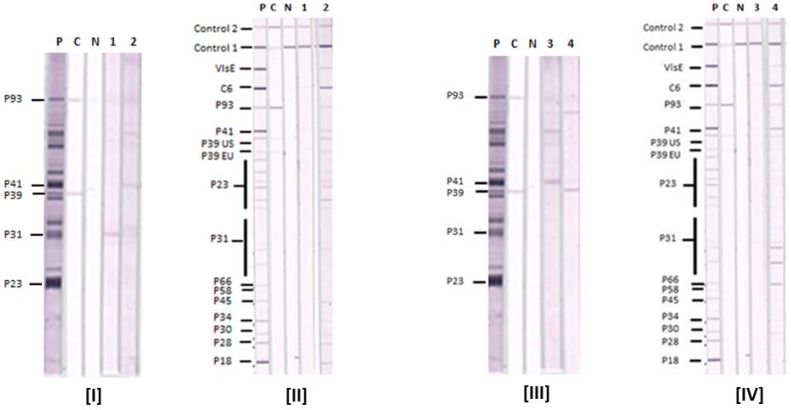
Comparison of Lyme IgM Western Blots and Lyme IgM Immunoblots. Results with serum samples (1–4) tested by Lyme IgM WB (I and III) and Lyme IgM IB (II and IV). P—positive control, C—calibrator and N—negative control. Control 1—conjugate control, and Control 2—serum control. The positions of target antigens in the IBs and WBs are shown. P39 EU—P39 from European BBsl species, P39 US—P39 from US BBsl species.

The results of Lyme IgM WB and IgM IB performed on four reference sera are shown in Figure 2. They show that the Lyme IgM IB is more sensitive than Lyme IgM WB with these four samples. Serum samples 1 and 3 are negative by either the CDC or in-house criteria in both Lyme IgM WB and IB. Serum samples 2 and 4 are negative in the Lyme IgM WB but positive in the Lyme IgM IB by both criteria. The IB-positive sera 2 and 4 also reacted with VlsE and C6 in the IBs. 

### 3.3. Lyme IgM and IgG Immunoblot and Western Blot Results with Known Lyme Disease-Positive Sera

The results obtained with the 26 known Lyme disease-positive sera among the 178 reference sera tested by Lyme IgM and IgG IBs as well as Lyme IgM and IgG WBs are presented in Table 2. They show that the both IgG and IgM Lyme IBs identified the same number or more positive sera overall than the corresponding IgG and IgM WBs by the in-house and CDC criteria. 

### 3.4. Lyme IgM and IgG Immunoblots and Western Blots Detecting Different Stages of Lyme Disease 

There were 17 sera from patients with confirmed Lyme disease among the 42 reference CDC samples (Table 1). Of these, the CDC had identified 10 patients with early Lyme disease, four with Lyme arthritis and three with Lyme neuroborreliosis (Table 3). The WB and IB results suggest that IgG and IgM IBs tend to be as good as or better than the corresponding IgG and IgM WBs for detecting the three stages of Lyme disease by either the in-house or CDC criteria for positivity. A high proportion of early Lyme disease and all three Lyme neuroborreliosis sera had detectable IgM antibodies in both IBs and WBs. When the two-tiered results were compared with Lyme IB read by in-house criteria, the specificity of the Lyme IB was comparable with the two-tiered testing (*p* = 1.0) and the sensitivity was improved significantly (*p* = 0.044). 

### 3.5. False Positive Reactions on Reference Sera in Lyme IgM and IgG Immunoblots and Western Blots 

The results obtained with the 152 reference sera that were known to be negative for Lyme disease (Table 1) when tested by Lyme IBs and WBs are presented in Table 4. The Lyme WBs with in-house criteria detected more false positive reactions with control sera from persons with syphilis and viral infections than the other tests performed, with the majority of these detecting IgM antibodies. 

### 3.6. Clinical Parameters of the Lyme Immunoblot and Western Blot Tests with Reference Sera

The results obtained with the 178 reference sera presented in Table 2 and Table 4 were used to calculate the sensitivity, specificity, PPV and NPV of serological detection regardless of disease stage with either IgG or IgM antibodies, with IgG antibodies and with IgM antibodies. The parameters obtained are shown in Table 5. The results suggest that because the 95% confidence intervals do not overlap, the specificity of detection with either IgG or IgM antibodies, IgG antibodies or IgM antibodies and PPV with either IgG or IgM antibodies is better in WBs on application of the CDC than the in-house criteria. The findings also show that the IBs tended to perform better than WBs with either in-house or CDC criteria for the detection of Lyme disease. This is particularly the case for specificity and PPV of either IgG or IgM antibodies or IgM antibodies by the in-house criteria where the 95% confidence intervals do not overlap between WBs and IBs. 

### 3.7. Comparison of Performance of Lyme IBs with Two-Tiered Testing (Whole-Cell EIA Followed by Confirmation with WBs)

The data (Table 5) demonstrates that the specificity of the Lyme IBs when read by in-house criteria is equivalent to two-tiered testing with WBs (*p* = 1.0) and the sensitivity is improved with Lyme IBs (*p* = 0.05). Interestingly with the CDC samples covering the full spectrum of disease, the sensitivity of the Lyme IBs was significantly improved (*p* = 0.044).

### 3.8. Antisera to TBRF Borrelia species B. hermsii and B. coriaceae Tested with Bbsl Antigens in Lyme IgG Immunoblots

**Figure 3:**
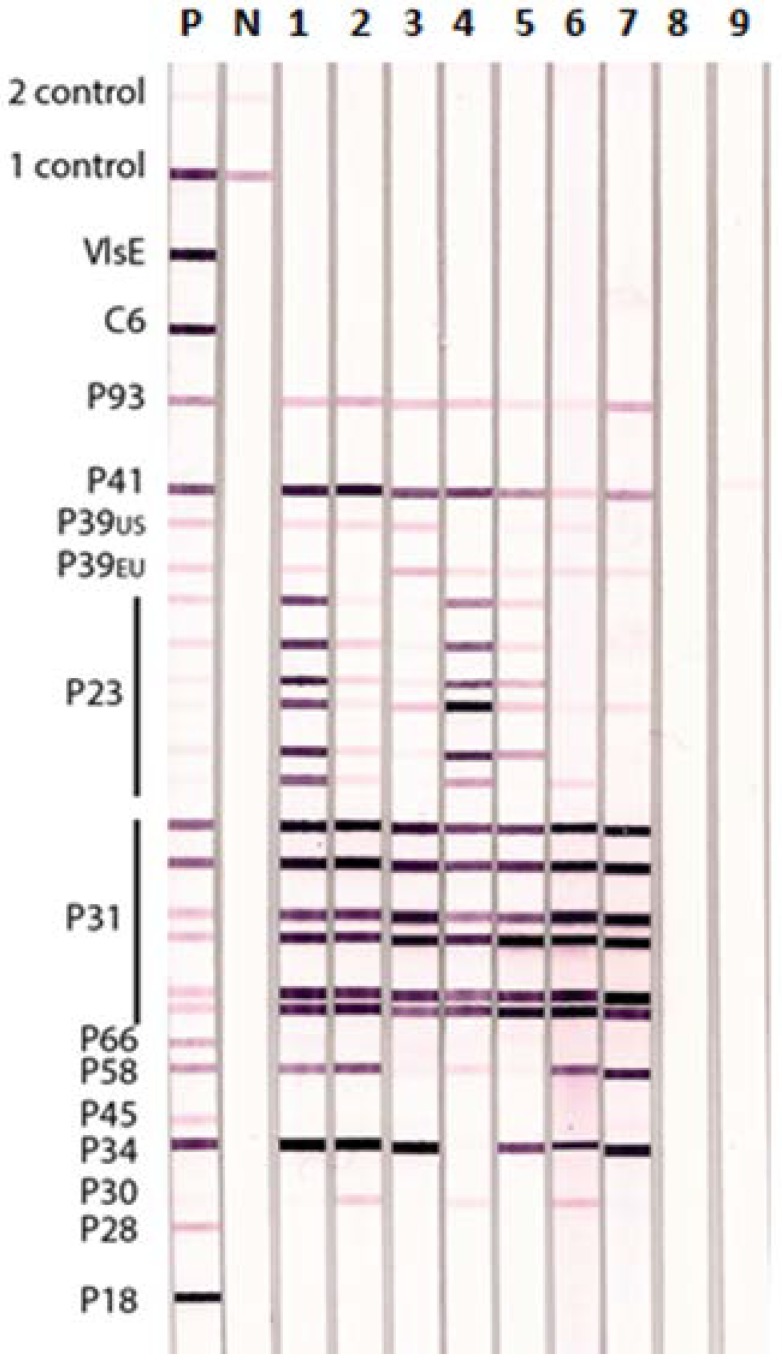
Lyme IgG Immunoblot Showing Reactions of Rabbit Antisera to Different BBsl and TBRF *Borrelia* species. Specific rabbit antisera produced against different BBsl (lanes 1–7) and TBRF (lanes 8 and 9). *Borrelia* species were tested individually on Lyme IgG IBs: Lane 1—*B. burgdorferi ss* B31, 2—*B. burgdorferi ss* 297, 3—*B. afzelii*, 4—*B. garinii*, 5—*B. californiensis*, 6—*B. spielmanii*, 7—*B. valensiana*, 8—*B. hermsii*, and 9—*B. coriaceae*. P—positive control human serum, N—negative control human serum, 1 Control—conjugate control, and 2 Control—serum control.

The results of Lyme IgG IBs performed with rabbit antisera raised against different BBsl species and TBRF *Borrelia* species are shown in Figure 3. All the rabbit anti- BBsl group sera were positive on the Lyme IgG IB whereas the rabbit antisera to the two TBRF *Borrelia* species, *B. hermsii* and *B. coriaceae*, only showed a weak cross-reaction with BBsl P41 (flagellin) in the Lyme IgG IB. Antisera against the different BBsl species showed variable strength of reaction with different BBsl antigens used in the IB. For example, the reaction with P23 (OspC) was more variable than that with P31 (OspA). None of the rabbit anti-BBsl sera reacted with VlsE or C6. 

## 4. Discussion

The two-tiered serological approach for laboratory diagnosis of Lyme disease [10,17] has continued to be widely used with EIA as the preferred first test followed by WB as the common second test. This two-step procedure was initiated because first-generation EIAs for the detection of anti-*Borrelia* antibodies lacked specificity. The inclusion of a second, more specific, serological method such as WB made it possible to exclude false-positive EIA samples. The shortcomings and advantages of this approach have been extensively examined [38,41,42,43]. The main drawbacks identified in such studies were the (i) relatively poor sensitivity in early disease [38,42,43]; (ii) persistence of antibodies even after the infection is cleared by treatment; (iii) less than optimal specificity of the WB used as a confirmatory test [41]; (iv) variability in sensitivity and specificity of EIAs and WBs employing recombinant target antigens for detection of anti-*Borrelia* antibodies [41] leading to unsatisfactory concordance in results between different laboratories using similar test formats and (v) possible subjectivity in scoring WB results. Nevertheless, the studies [38,41,42,43] highlight the continuing usefulness of the two-tiered diagnostic approach, whole-cell lysate EIA, followed by confirmation with WB (prepared from cell lysate); and using the CDC criteria for positive WBs because of the high specificity compared with other methods including those employing recombinant target antigens in EIA and WB. Our present findings also show that the CDC criteria for WBs yield better PPVs and similar NPVs to the in-house criteria for WBs. However our results, based on procedures to minimize or eliminate subjective assessment, suggest that the sensitivity, specificity, PPV and NPV values tend to be better with the newly developed IBs than WBs by either the CDC or in-house criteria for positivity. In addition, the results demonstrate that the specificity of the Lyme IBs is equivalent to two-tiered testing (using whole cell lysate EIA and WBs) with improved sensitivity (Table 5).

Seventeen of the 26 reference samples obtained from the CDC were from patients with Lyme disease and of these 10 were from patients with early Lyme disease as classified by the CDC. A high proportion of the 10 early Lyme sera had IgM antibodies that were detectable in WBs and IBs by either the CDC or the in-house criteria for positivity. This is consistent with the early formation of IgM antibodies during infection [10,17]. The nine Lyme positive proficiency test samples from the College of American Pathologists and the New York State Department of Health were not classified according to the stage of the Lyme disease. However all nine samples had IgM antibodies in WBs and IBs using either the CDC or the in-house criteria for positivity.

Because the precise time after infection when many positive reference sera were collected from patients with Lyme disease was not available, the presence of either IgM or IgG antibodies as well as IgM and IgG antibodies alone were separately analyzed for the clinical diagnostic parameters of sensitivity, specificity, PPV and NPV. The results obtained from the 178 reference serum samples show that IBs tend to have in all cases superior clinical diagnostic parameters to WBs for detecting BBsl-specific antibodies by either the current CDC or the in-house developed criteria for positivity. The decrease in sensitivity when only IgG antibodies are considered, compared with either IgG or IgM, may partly be due to some cases of early Lyme disease in the samples where IgG production lags behind IgM formation and partly due to the continued formation of specific IgM anti-BBsl antibodies in late disease as reported by others [44]. The tendency for a higher specificity in WBs by the in-house criteria when only IgG antibody responses are considered in comparison with either IgG or IgM antibodies may partly be due the prominent presence of cross-reacting IgM antibodies in viral infections and syphilis in the 74 reference samples from New York Biologics. The PPV of 83.9% with detection of either IgG or IgM antibodies in IBs read by the in-house criteria was obtained with 178 reference sera of which only 26 (or 14.6% prevalence) were from patients with Lyme disease. The PPV can be expected to improve when sera from patients who are suspected to have Lyme disease on clinical criteria are tested because of the expected higher disease prevalence in this population. Further studies on patient sera are therefore needed to fully evaluate the clinical diagnostic parameters of IBs described here.

A disadvantage with WBs is the presence of non-specific proteins migrating at the same positions as the specific antigens used for scoring WBs. An example of this is a binding of a non-specific protein of 31kDa at the same position as Osp A on WB strips [34]. Removing patients who showed a positive 31kDa band on WB but tested negative for antibodies to recombinant OspA antigen from the analysis improved the specificity to >97% for both IgM and IgG Lyme WBs [34]. The inclusion of purified recombinant OspA protein as antigen in Lyme IBs therefore maintains sensitivity but improves specificity of Lyme IB compared to Lyme WB. Thus with Lyme IB no further confirmatory testing for OspA antigen reactivity is required. Other advantages of IBs over WBs are that IBs permit selection of proteins of diagnostic importance, including those from different Bbsl species, and avoid variations due to genetic factors and culture conditions [12,34,35,36] that can affect the antigenicity of scored proteins in WBs.

The IBs showed that different Lyme-positive sera differentially recognized P23 and P31 from different BBsl species and P39 from US and European BBsl. This suggests that the use of recombinant proteins from several species of BBsl in the Lyme IBs contributes to their better diagnostic performance compared to Lyme WBs. However, other proteins used for scoring IBs show sequence homology among different BBsl species and are therefore expected to show varying degrees of antigenic cross-reactivity. Further studies with patient sera in conjunction with PCR-based tests for identifying species are required to determine whether IBs can help identify the BBsl species causing the infection.

The findings with rabbit antibodies reported here also demonstrate the potential of IBs for differentiating sera from patients with Lyme disease and TBRF. Whole cell lysates of *Borrelia* species causing Lyme disease and TBRF contain antigens that are shared between the two groups of *Borrelia* [15,16]. Based on the present findings that rabbit antisera to the two TBRF *Borrelia* species *B. hermsii* and *B. coriaceae* do not cross-react with the recombinant antigens used in Lyme IBs with the exception of a weak reaction with flagellin, it may be expected that sera from patients with *B. hermsii* and *B. coriaceae* infections will be similarly unreactive in Lyme IBs. However these predictions need to be confirmed in further studies. Our additional unpublished data suggest that it is possible to identify patients with mixed infections of BBsl species and TBRF *Borrelia* species and also clearly differentiate Lyme disease from TBRF in patients with either disease through the use of Lyme IBs and similar IBs developed for TBRF using TBRF *Borrelia*-specific recombinant antigens. 

A limitation of our study is that it is based on a collection of reference serum samples obtained from 26 patients with Lyme disease, but only 17 were characterized by stage of disease. Studies on a larger number of samples from patients with more clearly defined stages of this infection would be useful to better demonstrate the utility of Lyme IBs in laboratory evaluation of Lyme disease. Since 6.6% of Lyme disease patients can only be detected by the LM-PCR assay on urine and blood and not by the currently recommended WB serological assay [25], additional testing by the LM-PCR assay would also be useful in further evaluating Lyme IB tests. 

## Figures and Tables

**Table 1:** Reference Serum Samples and Expected Results for Lyme disease.

Source	Samples	Number of Samples	Expected Results for Lyme Disease	
			Positive	Negative
CDC	CDC—Set 1	10	5	5
CDC	CDC—Set 2	32	12	20
CAP and NYSH	Proficiency test (PT) samples	20	9	11
CAP and NYSH	Autoimmune diseases (22 with RA)	42	0	42
NYB	Virus infections	46	0	46
NYB	RPR + ve	28	0	28
**Total**		**178**	**26**	**152**

RA—rheumatoid arthritis; RPR—rapid plasma reagin test for syphilis; CAP—College of American Pathologists; NYB—New York Biologics, Southampton, NY; NYSH—New York State Department of Health.

**Table 2:** Reactivity of Known Lyme Disease-Positive Reference Sera in IgM and IgG Western Blots and Immunoblots.

Samples	Number of Known Positive Sera	EIA (+)	Positive						2-Tiered (CDC)	Positive					
			Lyme WB (In-House)			Lyme WB (CDC)				Lyme IB (In-House)			Lyme IB (CDC)		
			IgM	IgG	IgG or IgM	IgM	IgG	IgG or IgM		IgM	IgG	IgG or IgM	IgM	IgG	IgG or IgM
CDC-Set 1 *	5	4	2	4	4	2	3	4	4	3	5	5	2	4	4
CDC-Set 2 *	12	8	7	8	9	7	5	9	8	11	10	12	9	4	10
PT Samples	9	9	9	6	9	9	6	9	9	9	6	9	9	6	9
**Total Positive**	**26**	**21**	**18**	**18**	**22**	**18**	**14**	**22**	**21**	**23**	**21**	**26**	**20**	**14**	**23**

* CDC provided the samples and the EIA, Western Blot bands, Western Blot interpretation by CDC criteria and two-tiered results.

**Table 3:** IgM and IgG Western Blots and Immunoblots Results in Different Stages of Lyme disease.

Samples	Number of Known Positive Sera	EIA (+)	Positive						2-Tiered (CDC)	Positive					
			Lyme WB (In-House)			Lyme WB (CDC)				Lyme IB (In-House)			Lyme IB (CDC)		
			IgM	IgG	IgG or IgM	IgM	IgG	IgG or IgM		IgM	IgG	IgG or IgM	IgM	IgG	IgG or IgM
Early Lyme	10	6	6	5	6	5	1	6	5	8	8	10	7	1	7
Lyme Arthritis	4	4	0	4	4	0	4	4	4	3	4	4	2	4	4
Lyme Neuroborreliosis	3	3	3	3	3	3	3	3	3	3	3	3	3	3	3
**Total Positive**	**17**	**13**	**9**	**12**	**13**	**8**	**8**	**13**	**12**	**14**	**15**	**17**	**12**	**8**	**14**

CDC provided the samples and the EIA, Western Blot bands, Western Blot interpretation by CDC criteria and two-tiered results.

**Table 4:** IgM and IgG Western Blot and Immunoblot Results with Non-Lyme Disease Reference Sera.

Samples	Number of Known Positive Sera	EIA (+)	Positive						2-Tiered (CDC)	Positive					
			Lyme WB (In-House)			Lyme WB (CDC)				Lyme IB (In-House)			Lyme IB (CDC)		
			IgM	IgG	IgG or IgM	IgM	IgG	IgG or IgM		IgM	IgG	IgG or IgM	IgM	IgG	IgG or IgM
CDC—Set 1 *	5	0	0	1	1	0	0	0	0	0	0	0	0	0	0
CDC—Set 2 *	20	4	0	1	1	0	0	0	0	0	1	1	0	0	0
PT Samples	11	0	0	0	0	0	0	0	0	0	0	0	0	0	0
Autoimmune disease	42	0	0	0	0	0	0	0	0	0	0	0	0	0	0
Viral Infections	46	3	10	5	15	2	0	2	2	0	1	1	0	0	0
RPR +	28	11	6	3	7	1	0	1	1	0	2	2	0	1	1
**Total False Positive**		**18**	**16**	**10**	**24**	**3**	**0**	**3**	**3**	**0**	**4**	**4**	**0**	**1**	**1**
**Total True Negative**	**152**	**134**	**136**	**142**	**128**	**149**	**152**	**149**	**149**	**152**	**148**	**148**	**152**	**151**	**151**

* CDC provided the samples and the EIA, Western Blot bands, Western Blot interpretation by CDC criteria and two-tiered results.

**Table 5:** Clinical Parameters of the Serological Detection of Lyme disease by Western Blots and Immunoblots with the Reference Sera.

	WB In-House	WB CDC	2-Tiered CDC	IB In-House	IB CDC
Sensitivity IgG or IgM	84.6 (64.3–95.0)	84.6 (64.3–95.0)	80.8 (60.0–92.7)	100.0 (84.0–100)	88.5 (68.7–97.0)
Sensitivity IgG	69.2 (48.1–84.9)	53.8 (33.7–72.9)		80.8 (60.0–93.7)	57.7 (37.2–76.0)
Sensitivity IgM	75.0 (52.9–89.4)	69.2 (48.1–84.9)		88.5 (68.7–97.0)	73.1 (51.9–87.6)
Specificity IgG or IgM	84.2 (77.2–89.4)	98.0 (93.9–99.5)	98.0 (93.9–99.5)	96.7 (92.1–98.8)	99.3 (95.8–100)
Specificity IgG	93.4 (87.9–96.6)	100.0 (96.9–100)		97.4 (93.0–99.2)	99.3 (95.8–100)
Specificity IgM	89.5 (83.2–93.7)	98.0 (93.9–99.5)		100.0 (96.9–100)	100 (96.9–100)
PPV IgG or IgM	47.8 (33.1–62.9)	88.0 (67.7–96.8)	87.5 (66.6–96.7)	83.9 (65.5–93.9)	95.8 (76.9–99.8)
PPV IgG	64.3 (44.1–80.7)	100.0 (73.2–100)		84.0 (63.1–94.7)	93.8 (67.7–99.7)
PPV IgM	52.9 (35.4–69.8)	85.7 (62.6–96.2)		100.0 (82.2–100)	100.0 (79.1–100)
NPV IgG or IgM	97.0 (91.9–99.0)	97.4 (93.0–99.2)	96.8 (92.2–98.7)	100.0 (96.8–100)	98.1 (94.0–99.5)
NPV IgG	94.7 (89.4–97.5)	92.7 (87.3–96.0)		96.7 (92.1–98.8)	93.2 (87.9–96.4)
NPV IgM	95.8 (90.6–98.3)	94.9 (89.9–97.6)		98.1 (94.0–99.5)	95.6 (90.8–98.1)

Values are the percent expected values with its 95% confidence intervals in parentheses.
